# Big Data Analytics in Healthcare

**DOI:** 10.1155/2015/370194

**Published:** 2015-07-02

**Authors:** Ashwin Belle, Raghuram Thiagarajan, S. M. Reza Soroushmehr, Fatemeh Navidi, Daniel A. Beard, Kayvan Najarian

**Affiliations:** ^1^Emergency Medicine Department, University of Michigan, Ann Arbor, MI 48109, USA; ^2^University of Michigan Center for Integrative Research in Critical Care (MCIRCC), Ann Arbor, MI 48109, USA; ^3^Department of Molecular and Integrative Physiology, University of Michigan, Ann Arbor, MI 48109, USA; ^4^Department of Industrial and Operations Engineering, University of Michigan, Ann Arbor, MI 48109, USA

## Abstract

The rapidly expanding field of big data analytics has started to play a pivotal role in the evolution of healthcare practices and research. It has provided tools to accumulate, manage, analyze, and assimilate large volumes of disparate, structured, and unstructured data produced by current healthcare systems. Big data analytics has been recently applied towards aiding the process of care delivery and disease exploration. However, the adoption rate and research development in this space is still hindered by some fundamental problems inherent within the big data paradigm. In this paper, we discuss some of these major challenges with a focus on three upcoming and promising areas of medical research: image, signal, and genomics based analytics. Recent research which targets utilization of large volumes of medical data while combining multimodal data from disparate sources is discussed. Potential areas of research within this field which have the ability to provide meaningful impact on healthcare delivery are also examined.

## 1. Introduction

The concept of “big data” is not new; however the way it is defined is constantly changing. Various attempts at defining big data essentially characterize it as a collection of data elements whose size, speed, type, and/or complexity require one to seek, adopt, and invent new hardware and software mechanisms in order to successfully store, analyze, and visualize the data [1–3]. Healthcare is a prime example of how the three Vs of data, velocity (speed of generation of data), variety, and volume [4], are an innate aspect of the data it produces. This data is spread among multiple healthcare systems, health insurers, researchers, government entities, and so forth. Furthermore, each of these data repositories is siloed and inherently incapable of providing a platform for global data transparency. To add to the three Vs, the veracity of healthcare data is also critical for its meaningful use towards developing translational research.

Despite the inherent complexities of healthcare data, there is potential and benefit in developing and implementing big data solutions within this realm. A report by McKinsey Global Institute suggests that if US healthcare were to use big data creatively and effectively, the sector could create more than $300 billion in value every year. Two-thirds of the value would be in the form of reducing US healthcare expenditure [5]. Historical approaches to medical research have generally focused on the investigation of disease states based on the changes in physiology in the form of a confined view of certain singular modality of data [6]. Although this approach to understanding diseases is essential, research at this level mutes the variation and interconnectedness that define the true underlying medical mechanisms [7]. After decades of technological laggard, the field of medicine has begun to acclimatize to today's digital data age. New technologies make it possible to capture vast amounts of information about each individual patient over a large timescale. However, despite the advent of medical electronics, the data captured and gathered from these patients has remained vastly underutilized and thus wasted.

Important physiological and pathophysiological phenomena are concurrently manifest as changes across multiple clinical streams. This results from strong coupling among different systems within the body (e.g., interactions between heart rate, respiration, and blood pressure) thereby producing potential markers for clinical assessment. Thus, understanding and predicting diseases require an aggregated approach where structured and unstructured data stemming from a myriad of clinical and nonclinical modalities are utilized for a more comprehensive perspective of the disease states. An aspect of healthcare research that has recently gained traction is in addressing some of the growing pains in introducing concepts of big data analytics to medicine. Researchers are studying the complex nature of healthcare data in terms of both characteristics of the data itself and the taxonomy of analytics that can be meaningfully performed on them.

In this paper, three areas of big data analytics in medicine are discussed. These three areas do not comprehensively reflect the application of big data analytics in medicine; instead they are intended to provide a perspective of broad, popular areas of research where the concepts of big data analytics are currently being applied.

*Image Processing.* Medical images are an important source of data frequently used for diagnosis, therapy assessment and planning [8]. Computed tomography (CT), magnetic resonance imaging (MRI), X-ray, molecular imaging, ultrasound, photoacoustic imaging, fluoroscopy, positron emission tomography-computed tomography (PET-CT), and mammography are some of the examples of imaging techniques that are well established within clinical settings. Medical image data can range anywhere from a few megabytes for a single study (e.g., histology images) to hundreds of megabytes per study (e.g., thin-slice CT studies comprising upto 2500+ scans per study [9]). Such data requires large storage capacities if stored for long term. It also demands fast and accurate algorithms if any decision assisting automation were to be performed using the data. In addition, if other sources of data acquired for each patient are also utilized during the diagnoses, prognosis, and treatment processes, then the problem of providing cohesive storage and developing efficient methods capable of encapsulating the broad range of data becomes a challenge. 

*Signal Processing*. Similar to medical images, medical signals also pose volume and velocity obstacles especially during continuous, high-resolution acquisition and storage from a multitude of monitors connected to each patient. However, in addition to the data size issues, physiological signals also pose complexity of a spatiotemporal nature. Analysis of physiological signals is often more meaningful when presented along with situational context awareness which needs to be embedded into the development of continuous monitoring and predictive systems to ensure its effectiveness and robustness.

Currently healthcare systems use numerous disparate and continuous monitoring devices that utilize singular physiological waveform data or discretized vital information to provide alert mechanisms in case of overt events. However, such uncompounded approaches towards development and implementation of alarm systems tend to be unreliable and their sheer numbers could cause* “alarm fatigue”* for both care givers and patients [10–12]. In this setting, the ability to discover new medical knowledge is constrained by prior knowledge that has typically fallen short of maximally utilizing high-dimensional time series data. The reason that these alarm mechanisms tend to fail is primarily because these systems tend to rely on single sources of information while lacking context of the patients' true physiological conditions from a broader and more comprehensive viewpoint. Therefore, there is a need to develop improved and more comprehensive approaches towards studying interactions and correlations among multimodal clinical time series data. This is important because studies continue to show that humans are poor in reasoning about changes affecting more than two signals [13–15]. 

*Genomics*. The cost to sequence the human genome (encompassing 30,000 to 35,000 genes) is rapidly decreasing with the development of high-throughput sequencing technology [16, 17]. With implications for current public health policies and delivery of care [18, 19], analyzing genome-scale data for developing actionable recommendations in a timely manner is a significant challenge to the field of computational biology. Cost and time to deliver recommendations are crucial in a clinical setting. Initiatives tackling this complex problem include tracking of 100,000 subjects over 20 to 30 years using the predictive, preventive, participatory, and personalized health, refered to as P4, medicine paradigm [20–22] as well as an integrative personal omics profile [23]. The P4 initiative is using a system approach for (i) analyzing genome-scale datasets to determine disease states, (ii) moving towards blood based diagnostic tools for continuous monitoring of a subject, (iii) exploring new approaches to drug target discovery, developing tools to deal with big data challenges of capturing, validating, storing, mining, integrating, and finally (iv) modeling data for each individual. The integrative personal omics profile (iPOP) combines physiological monitoring and multiple high-throughput methods for genome sequencing to generate a detailed health and disease states of a subject [23]. Ultimately, realizing actionable recommendations at the clinical level remains a grand challenge for this field [24, 25]. Utilizing such high density data for exploration, discovery, and clinical translation demands novel big data approaches and analytics.

Despite the enormous expenditure consumed by the current healthcare systems, clinical outcomes remain suboptimal, particularly in the USA, where 96 people per 100,000 die annually from conditions considered treatable [26]. A key factor attributed to such inefficiencies is the inability to effectively gather, share, and use information in a more comprehensive manner within the healthcare systems [27]. This is an opportunity for big data analytics to play a more significant role in aiding the exploration and discovery process, improving the delivery of care, helping to design and plan healthcare policy, providing a means for comprehensively measuring, and evaluating the complicated and convoluted healthcare data. More importantly, adoption of insights gained from big data analytics has the potential to save lives, improve care delivery, expand access to healthcare, align payment with performance, and help curb the vexing growth of healthcare costs.

## 2. Medical Image Processing from Big Data Point of View

Medical imaging provides important information on anatomy and organ function in addition to detecting diseases states. Moreover, it is utilized for organ delineation, identifying tumors in lungs, spinal deformity diagnosis, artery stenosis detection, aneurysm detection, and so forth. In these applications, image processing techniques such as enhancement, segmentation, and denoising in addition to machine learning methods are employed. As the size and dimensionality of data increase, understanding the dependencies among the data and designing efficient, accurate, and computationally effective methods demand new computer-aided techniques and platforms. The rapid growth in the number of healthcare organizations as well as the number of patients has resulted in the greater use of computer-aided medical diagnostics and decision support systems in clinical settings. Many areas in health care such as diagnosis, prognosis, and screening can be improved by utilizing computational intelligence [28]. The integration of computer analysis with appropriate care has potential to help clinicians improve diagnostic accuracy [29]. The integration of medical images with other types of electronic health record (EHR) data and genomic data can also improve the accuracy and reduce the time taken for a diagnosis.

In the following, data produced by imaging techniques are reviewed and applications of medical imaging from a big data point of view are discussed.

### 2.1. Data Produced by Imaging Techniques

Medical imaging encompasses a wide spectrum of different image acquisition methodologies typically utilized for a variety of clinical applications. For example, visualizing blood vessel structure can be performed using magnetic resonance imaging (MRI), computed tomography (CT), ultrasound, and photoacoustic imaging [30]. From a data dimension point of view, medical images might have 2, 3, and four dimensions. Positron emission tomography (PET), CT, 3D ultrasound, and functional MRI (fMRI) are considered as multidimensional medical data. Modern medical image technologies can produce high-resolution images such as respiration-correlated or “four-dimensional” computed tomography (4D CT) [31]. Higher resolution and dimensions of these images generate large volumes of data requiring high performance computing (HPC) and advanced analytical methods. For instance, microscopic scans of a human brain with high resolution can require 66TB of storage space [32]. Although the volume and variety of medical data make its analysis a big challenge, advances in medical imaging could make individualized care more practical [33] and provide quantitative information in variety of applications such as disease stratification, predictive modeling, and decision making systems. In the following we refer to two medical imaging techniques and one of their associated challenges.

Molecular imaging is a noninvasive technique of cellular and subcellular events [34] which has the potential for clinical diagnosis of disease states such as cancer. However, in order to make it clinically applicable for patients, the interaction of radiology, nuclear medicine, and biology is crucial [35] that could complicate its automated analysis.

Microwave imaging is an emerging methodology that could create a map of electromagnetic wave scattering arising from the contrast in the dielectric properties of different tissues [36]. It has both functional and physiological information encoded in the dielectric properties which can help differentiate and characterize different tissues and/or pathologies [37]. However, microwaves have scattering behavior that makes retrieval of information a challenging task.

The integration of images from different modalities and/or other clinical and physiological information could improve the accuracy of diagnosis and outcome prediction of disease. Liebeskind and Feldmann explored advances in neurovascular imaging and the role of multimodal CT or MRI including angiography and perfusion imaging on evaluating the brain vascular disorder and achieving precision medicine [33]. Delayed enhanced MRI has been used for exact assessment of myocardial infarction scar [38]. For this kind of disease, electroanatomic mapping (EAM) can help in identifying the subendocardial extension of infarct. The role of evaluating both MRI and CT images to increase the accuracy of diagnosis in detecting the presence of erosions and osteophytes in the temporomandibular joint (TMJ) has been investigated by Hussain et al. [39]. According to this study simultaneous evaluation of all the available imaging techniques is an unmet need.

Advanced Multimodal Image-Guided Operating (AMIGO) suite has been designed which has angiographic X-ray system, MRI, 3D ultrasound, and PET/CT imaging in the operating room (OR). This system has been used for cancer therapy and showed the improvement in localization and targeting an individual's diseased tissue [40].

Besides the huge space required for storing all the data and their analysis, finding the map and dependencies among different data types are challenges for which there is no optimal solution yet.

### 2.2. Methods

The volume of medical images is growing exponentially. For instance, ImageCLEF medical image dataset contained around 66,000 images between 2005 and 2007 while just in the year of 2013 around 300,000 images were stored everyday [41]. In addition to the growing volume of images, they differ in modality, resolution, dimension, and quality which introduce new challenges such as data integration and mining specially if multiple datasets are involved. Compared to the volume of research that exists on single modal medical image analysis, there is considerably lesser number of research initiatives on multimodal image analysis.

When utilizing data at a local/institutional level, an important aspect of a research project is on how the developed system is evaluated and validated. Having annotated data or a structured method to annotate new data is a real challenge. This becomes even more challenging when large-scale data integration from multiple institutions are taken into account. As an example, for the same applications (e.g., traumatic brain injury) and the same modality (e.g., CT), different institutes might use different settings in image acquisitions which makes it hard to develop unified annotation or analytical methods for such data. In order to benefit the multimodal images and their integration with other medical data, new analytical methods with real-time feasibility and scalability are required. In the following we look at analytical methods that deal with some aspects of big data.

#### 2.2.1. Analytical Methods

The goal of medical image analytics is to improve the interpretability of depicted contents [8]. Many methods and frameworks have been developed for medical image processing. However, these methods are not necessarily applicable for big data applications.

One of the frameworks developed for analyzing and transformation of very large datasets is Hadoop that employs MapReduce [42, 43]. MapReduce is a programming paradigm that provides scalability across many servers in a Hadoop cluster with a broad variety of real-world applications [44–46]. However, it does not perform well with input-output intensive tasks [47]. MapReduce framework has been used in [47] to increase the speed of three large-scale medical image processing use-cases, (i) finding optimal parameters for lung texture classification by employing a well-known machine learning method, support vector machines (SVM), (ii) content-based medical image indexing, and (iii) wavelet analysis for solid texture classification. In this framework, a cluster of heterogeneous computing nodes with a maximum of 42 concurrent map tasks was set up and the speedup around 100 was achieved. In other words, total execution time for finding optimal SVM parameters was reduced from about 1000 h to around 10 h. Designing a fast method is crucial in some applications such as trauma assessment in critical care where the end goal is to utilize such imaging techniques and their analysis within what is considered as a golden-hour of care [48]. Therefore, execution time or real-time feasibility of developed methods is of importance. Accuracy is another factor that should be considered in designing an analytical method. Finding dependencies among different types of data could help improve the accuracy. For instance, a hybrid machine learning method has been developed in [49] that classifies schizophrenia patients and healthy controls using fMRI images and single nucleotide polymorphism (SNP) data [49]. The authors reported an accuracy of 87% classification, which would not have been as high if they had used just fMRI images or SNP alone. del Toro and Muller have compared some organ segmentation methods when data is considered as big data. They have proposed a method that incorporates both local contrast of the image and atlas probabilistic information [50]. An average of 33% improvement has been achieved compared to using only atlas information. Tsymbal et al. have designed a clinical decision support system that exploits discriminative distance learning with significantly lower computational complexity compared to classical alternatives and hence this system is more scalable to retrieval [51]. A computer-aided decision support system was developed by Chen et al. [52] that could assist physicians to provide accurate treatment planning for patients suffering from traumatic brain injury (TBI). In this method, patient's demographic information, medical records, and features extracted from CT scans were combined to predict the level of intracranial pressure (ICP). The accuracy, sensitivity, and specificity were reported to be around 70.3%, 65.2%, and 73.7%, respectively. In [53], molecular imaging and its impact on cancer detection and cancer drug improvement are discussed. The proposed technology is designed to aid in early detection of cancer by integrating molecular and physiological information with anatomical information. Using this imaging technique for patients with advanced ovarian cancer, the accuracy of the predictor of response to a special treatment has been increased compared to other clinical or histopathologic criteria. A hybrid digital-optical correlator (HDOC) has been designed to speed up the correlation of images [54]. HDOC can be employed to compare images in the absence of coordinate matching or georegistration. In this multichannel method, the computation is performed in the storage medium which is a volume holographic memory which could help HDOC to be applicable in the area of big data analytics [54].

#### 2.2.2. Collecting, Sharing, and Compressing Methods

In addition to developing analytical methods, efforts have been made for collecting, compressing, sharing, and anonymizing medical data. One example is iDASH (integrating data for analysis, anonymization, and sharing) which is a center for biomedical computing [55]. It focuses on algorithms and tools for sharing data in a privacy-preserving manner. The goal of iDASH is to bring together a multi-institutional team of quantitative scientists to develop algorithms and tools, services, and a biomedical cyber infrastructure to be used by biomedical and behavioral researchers [55]. Another example of a similar approach is Health-e-Child consortium of 14 academic, industry, and clinical partners with the aim of developing an integrated healthcare platform for European paediatrics [51].

Based on the Hadoop platform, a system has been designed for exchanging, storing, and sharing electronic medical records (EMR) among different healthcare systems [56]. This system can also help users retrieve medical images from a database. Medical data has been investigated from an acquisition point of view where patients' vital data is collected through a network of sensors [57]. This system delivers data to a cloud for storage, distribution, and processing. A prototype system has been implemented in [58] to handle standard store/query/retrieve requests on a database of Digital Imaging and Communications in Medicine (DICOM) images. This system uses Microsoft Windows Azure as a cloud computing platform.

When dealing with a very large volume of data, compression techniques can help overcome data storage and network bandwidth limitations. Many methods have been developed for medical image compression. However, there are a few methods developed for big data compression. A method has been designed to compress both high-throughput sequencing dataset and the data generated from calculation of log-odds of probability error for each nucleotide and the maximum compression ratios of 400 and 5 have been achieved, respectively [55]. This dataset has medical and biomedical data including genotyping, gene expression, proteomic measurements with demographics, laboratory values, images, therapeutic interventions, and clinical phenotypes for Kawasaki Disease (KD). By illustrating the data with a graph model, a framework for analyzing large-scale data has been presented [59]. For this model, the fundamental signal processing techniques such as filtering and Fourier transform were implemented. In [60], the application of simplicity and power (SP) theory of intelligence in big data has been investigated. The goal of SP theory is to simplify and integrate concepts from multiple fields such as artificial intelligence, mainstream computing, mathematics, and human perception and cognition that can be observed as a brain-like system [60]. The proposed SP system performs lossless compression through the matching and unification of patterns. However, this system is still in the design stage and cannot be supported by today's technologies.

There are some limitations in implementing the application-specific compression methods on both general-purpose processors and parallel processors such as graphics processing units (GPUs) as these algorithms need highly variable control and complex bit manipulations which are not well suited to GPUs and pipeline architectures. To overcome this limitation, an FPGA implementation was proposed for LZ-factorization which decreases the computational burden of the compression algorithm [61]. A lossy image compression has been introduced in [62] that reshapes the image in such a way that if the image is uniformly sampled, sharp features have a higher sampling density than the coarse ones. This method is claimed to be applicable for big data compression. However, for medical applications lossy methods are not applicable in most cases as fidelity is important and information must be preserved.

These techniques are among a few techniques that have been either designed as prototypes or developed with limited applications. Developing methods for processing/analyzing a broad range and large volume of data with acceptable accuracy and speed is still critical. In Table 1, we summarize the challenges facing medical image processing. When dealing with big data, these challenges seemed to be more serious and on the other hand analytical methods could benefit the big data to handle them.

## 3. Medical Signal Analytics

Telemetry and physiological signal monitoring devices are ubiquitous. However, continuous data generated from these monitors have not been typically stored for more than a brief period of time, thereby neglecting extensive investigation into generated data. However, in the recent past, there has been an increase in the attempts towards utilizing telemetry and continuous physiological time series monitoring to improve patient care and management [77–80].

**Figure 1:**
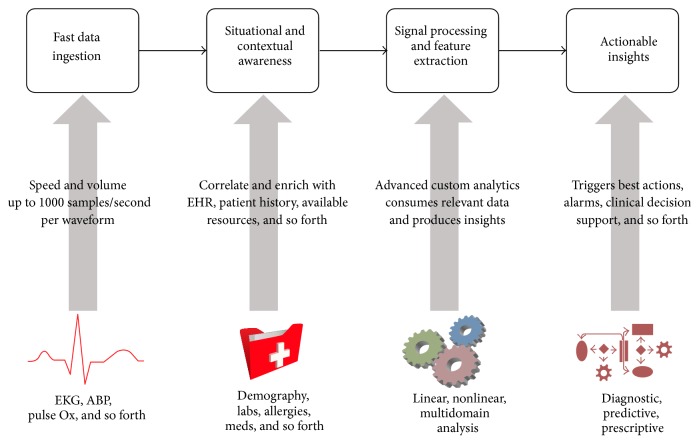
Generalized analytic workflow using streaming healthcare data.

Streaming data analytics in healthcare can be defined as a systematic use of continuous waveform (signal varying against time) and related medical record information developed through applied analytical disciplines (e.g., statistical, quantitative, contextual, cognitive, and predictive) to drive decision making for patient care. The analytics workflow of real-time streaming waveforms in clinical settings can be broadly described using Figure 1. Firstly, a platform for streaming data acquisition and ingestion is required which has the bandwidth to handle multiple waveforms at different fidelities. Integrating these dynamic waveform data with static data from the EHR is a key component to provide situational and contextual awareness for the analytics engine. Enriching the data consumed by analytics not only makes the system more robust, but also helps balance the sensitivity and specificity of the predictive analytics. The specifics of the signal processing will largely depend on the type of disease cohort under investigation. A variety of signal processing mechanisms can be utilized to extract a multitude of target features which are then consumed by a pretrained machine learning model to produce an actionable insight. These actionable insights could either be diagnostic, predictive, or prescriptive. These insights could further be designed to trigger other mechanisms such as alarms and notification to physicians.

Harmonizing such continuous waveform data with discrete data from other sources for finding necessary patient information and conducting research towards development of next generation diagnoses and treatments can be a daunting task [81]. For bed-side implementation of such systems in clinical environments, there are several technical considerations and requirements that need to be designed and implemented at system, analytic, and clinical levels. The following subsections provide an overview of different challenges and existing approaches in the development of monitoring systems that consume both high fidelity waveform data and discrete data from noncontinuous sources.

### 3.1. Data Acquisition

Historically streaming data from continuous physiological signal acquisition devices was rarely stored. Even if the option to store this data were available, the length of these data captures was typically short and downloaded only using proprietary software and data formats provided by the device manufacturers. Although most major medical device manufactures are now taking steps to provide interfaces to access live streaming data from their devices, such data in motion very quickly poses archetypal big data challenges. The fact that there are also governance challenges such as lack of data protocols, lack of data standards, and data privacy issues is adding to this. On the other side there are many challenges within the healthcare systems such as network bandwidth, scalability, and cost that have stalled the widespread adoption of such streaming data collection [82–84]. This has allowed way for system-wide projects which especially cater to medical research communities [77, 79, 80, 85–93].

Research community has interest in consuming data captured from live monitors for developing continuous monitoring technologies [94, 95]. There have been several indigenous and off-the-shelf efforts in developing and implementing systems that enable such data capture [85, 96–99]. There are also products being developed in the industry that facilitate device manufacturer agnostic data acquisition from patient monitors across healthcare systems.

### 3.2. Data Storage and Retrieval

With large volumes of streaming data and other patient information that can be gathered from clinical settings, sophisticated storage mechanisms of such data are imperative. Since storing and retrieving can be computational and time expensive, it is key to have a storage infrastructure that facilitates rapid data pull and commits based on analytic demands.

With its capability to store and compute large volumes of data, usage of systems such as Hadoop, MapReduce, and MongoDB [100, 101] is becoming much more common with the healthcare research communities. MongoDB is a free cross-platform document-oriented database which eschews traditional table-based relational database. Typically each health system has its own custom relational database schemas and data models which inhibit interoperability of healthcare data for multi-institutional data sharing or research studies. Furthermore, given the nature of traditional databases integrating data of different types such as streaming waveforms and static EHR data is not feasible. This is where MongoDB and other document-based databases can provide high performance, high availability, and easy scalability for the healthcare data needs [102, 103]. Apache Hadoop is an open source framework that allows for the distributed processing of large datasets across clusters of computers using simple programming models. It is a highly scalable platform which provides a variety of computing modules such as MapReduce and Spark. For performing analytics on continuous telemetry waveforms, a module like Spark is especially useful since it provides capabilities to ingest and compute on streaming data along with machine learning and graphing tools. Such technologies allow researchers to utilize data for both real-time as well as retrospective analysis, with the end goal to translate scientific discovery into applications for clinical settings in an effective manner.

### 3.3. Data Aggregation

Integration of disparate sources of data, developing consistency within the data, standardization of data from similar sources, and improving the confidence in the data especially towards utilizing automated analytics are among challenges facing data aggregation in healthcare systems [104]. Medical data can be complex in nature as well as being interconnected and interdependent; hence simplification of this complexity is important. Medical data is also subject to the highest level of scrutiny for privacy and provenance from governing bodies, therefore developing secure storage, access, and use of the data is very important [105].

Analysis of continuous data heavily utilizes the information in time domain. However, static data does not always provide true time context and, hence, when combining the waveform data with static electronic health record data, the temporal nature of the time context during integration can also add significantly to the challenges. There are considerable efforts in compiling waveforms and other associated electronic medical information into one cohesive database that are made publicly available for researchers worldwide [106, 107]. For example, MIMIC II [108, 109] and some other datasets included in Physionet [96] provide waveforms and other clinical data from a wide variety of actual patient cohorts.

### 3.4. Signal Analytics Using Big Data

Research in signal processing for developing big data based clinical decision support systems (CDSSs) is getting more prevalent [110]. In fact organizations such as the Institution of Medicine have long advocated use of health information technology including CDSS to improve care quality [111]. CDSSs provide medical practitioners with knowledge and patient-specific information, intelligently filtered and presented at appropriate times, to improve the delivery of care [112].

A vast amount of data in short periods of time is produced in intensive care units (ICU) where a large volume of physiological data is acquired from each patient. Hence, the potential for developing CDSS in an ICU environment has been recognized by many researchers. A scalable infrastructure for developing a patient care management system has been proposed which combines static data and stream data monitored from critically ill patients in the ICU for data mining and alerting medical staff of critical events in real time [113]. Similarly, Bressan et al. developed an architecture specialized for a neonatal ICU which utilized streaming data from infusion pumps, EEG monitors, cerebral oxygenation monitors, and so forth to provide clinical decision support [114]. A clinical trial is currently underway which extracts biomarkers through signal processing from heart and respiratory waveforms in real time to test whether maintaining stable heart rate and respiratory rate variability throughout the spontaneous breathing trials, administered to patients before extubation, may predict subsequent successful extubation [115]. An animal study shows how acquisition of noninvasive continuous data such as tissue oxygenation, fluid content, and blood flow can be used as indicators of soft tissue healing in wound care [78]. Electrocardiogrpah parameters from telemetry along with demographic information including medical history, ejection fraction, laboratory values, and medications have been used to develop an inhospital early detection system for cardiac arrest [116].

A study presented by Lee and Mark uses the MIMIC II database to prompt therapeutic intervention to hypotensive episodes using cardiac and blood pressure time series data [117]. Another study shows the use of physiological waveform data along with clinical data from the MIMIC II database for finding similarities among patients within the selected cohorts [118]. This similarity can potentially help care givers in the decision making process while utilizing outcomes and treatments knowledge gathered from similar disease cases from the past. A combination of multiple waveform information available in the MIMIC II database is utilized to develop early detection of cardiovascular instability in patients [119]. Many types of physiological data captured in the operative and preoperative care settings and how analytics can consume these data to help continuously monitor the status of the patients during, before and after surgery, are described in [120]. The potential of developing data fusion based machine learning models which utilizes biomarkers from breathomics (metabolomics study of exhaled air) as a diagnostic tool is demonstrated in [121].

Research in neurology has shown interest in electrophysiologic monitoring of patients to not only examine complex diseases under a new light but also develop next generation diagnostics and therapeutic devices. An article focusing on neurocritical care explores the different physiological monitoring systems specifically developed for the care of patients with disorders who require neurocritical care [122]. The authors of this article do not make specific recommendations about treatment, imaging, and intraoperative monitoring; instead they examine the potentials and implications of neuromonitoring with differeing quality of data and also provide guidance on developing research and application in this area. The development of multimodal monitoring for traumatic brain injury patients and individually tailored, patient specific care are examined in [123]. Zanatta et al. have investigated whether multimodal brain monitoring performed with TCD, EEG, and SEPs reduces the incidence of major neurologic complications in patients who underwent cardiac surgery. The authors evaluated whether the use of multimodal brain monitoring shortened the duration of mechanical ventilation required by patients as well as ICU and healthcare stays. The concepts of multimodal monitoring for secondary brain injury in neurocritical care as well as outline initial and future approaches using informatics tools for understanding and applying such data towards clinical care are described in [124].

As complex physiological monitoring devices are getting smaller, cheaper, and more portable, personal monitoring devices are being used outside of clinical environments by both patients and enthusiasts alike. However, similar to clinical applications, combining information simultaneously collected from multiple portable devices can become challenging. Pantelopoulos and Bourbakis discussed the research and development of wearable biosensor systems and identified the advantages and shortcomings in this area of study [125]. Similarly, portable and connected electrocardiogram, blood pressure and body weight devices are used to set up a network based study of telemedicine [126]. The variety of fixed as well as mobile sensors available for data mining in the healthcare sector and how such data can be leveraged for developing patient care technologies are surveyed in [127].

## 4. Big Data Applications in Genomics

The advent of high-throughput sequencing methods has enabled researchers to study genetic markers over a wide range of population [22, 128], improve efficiency by more than five orders of magnitude since sequencing of the human genome was completed [129], and associate genetic causes of the phenotype in disease states [130]. Genome-wide analysis utilizing microarrays has been successful in analyzing traits across a population and contributed successfully in treatments of complex diseases such as Crohn's disease and age-related muscular degeneration [130].

Analytics of high-throughput sequencing techniques in genomics is an inherently big data problem as the human genome consists of 30,000 to 35,000 genes [16, 17]. Initiatives are currently being pursued over the timescale of years to integrate clinical data from the genomic level to the physiological level of a human being [22, 23]. These initiatives will help in delivering personalized care to each patient. Delivering recommendations in a clinical setting requires fast analysis of genome-scale big data in a reliable manner. This field is still in a nascent stage with applications in specific focus areas, such as cancer [131–134], because of cost, time, and labor intensive nature of analyzing this big data problem.

Big data applications in genomics cover a wide variety of topics. Here we focus on pathway analysis, in which functional effects of genes differentially expressed in an experiment or gene set of particular interest are analyzed, and the reconstruction of networks, where the signals measured using high-throughput techniques are analyzed to reconstruct underlying regulatory networks. These networks influence numerous cellular processes which affect the physiological state of a human being [135].

### 4.1. Pathway Analysis

Resources for inferring functional effects for “-omics” big data are largely based on statistical associations between observed gene expression changes and predicted functional effects. Experiment and analytical practices lead to error as well as batch effects [136, 137]. Interpretation of functional effects has to incorporate continuous increases in available genomic data and corresponding annotation of genes [25]. There are variety of tools, but no “gold standard” for functional pathway analysis of high-throughput genome-scale data [138]. Three generations of methods used for pathway analysis [25] are described as follows.

The first generation encompasses overrepresentation analysis approaches that determine the fraction of genes in a particular pathway found among the genes which are differentially expressed [25]. Examples of the first generation tools are Onto-Express [139, 140], GoMiner [142], and ClueGo [144]. The second generation includes functional class scoring approaches which incorporate expression level changes in individual genes as well as functionally similar genes [25]. GSEA [146] is a popular tool that belongs to the second generation of pathway analysis. The third generation includes pathway topology based tools which are publicly available pathway knowledge databases with detailed information of gene products interactions: how specific gene products interact with each other and the location where they interact [25]. Pathway-Express [148] is an example of a third generation tool that combines the knowledge of differentially expressed genes with biologically meaningful changes on a given pathway to perform pathway analysis.

### 4.2. Reconstruction of Regulatory Networks

Pathway analysis approaches do not attempt to make sense of high-throughput big data in biology as arising from the integrated operation of a dynamical system [25]. There are multiple approaches to analyzing genome-scale data using a dynamical system framework [135, 152, 159]. Due to the breadth of the field, in this section we mainly focus on techniques to infer network models from biological big data. Applications developed for network inference in systems biology for big data applications can be split into two broad categories consisting of reconstruction of metabolic networks and gene regulatory networks [135]. Various approaches of network inference vary in performance, and combining different approaches has shown to produce superior predictions [152, 160].

Reconstruction of metabolic networks has advanced in last two decades. One objective is to develop an understanding of organism-specific metabolism through reconstruction of metabolic networks by integrating genomics, transcriptomics, and proteomics high-throughput sequencing techniques [150, 161–167]. Constraint-based methods are widely applied to probe the genotype-phenotype relationship and attempt to overcome the limited availability of kinetic constants [168, 169]. There are multitude of challenges in terms of analyzing genome-scale data including the experiment and inherent biological noise, differences among experimental platforms, and connecting gene expression to reaction flux used in constraint-based methods [170, 171].

Available reconstructed metabolic networks include Recon 1 [161], Recon 2 [150], SEED [163], IOMA [165], and MADE [172]. Recon 2 (an improvement over Recon 1) is a model to represent human metabolism and incorporates 7,440 reactions involving 5,063 metabolites. Recon 2 has been expanded to account for known drugs for drug target prediction studies [151] and to study off-target effects of drugs [173].

Reconstruction of gene regulatory networks from gene expression data is another well developed field. Network inference methods can be split into five categories based on the underlying model in each case: regression, mutual information, correlation, Boolean regulatory networks, and other techniques [152]. Over 30 inference techniques were assessed after DREAM5 challenge in 2010 [152]. Performance varied within each category and there was no category found to be consistently better than the others. Different methods utilize different information available in experiments which can be in the form of time series, drug perturbation experiments, gene knockouts, and combinations of experimental conditions. A tree-based method (using ensembles of regression trees) [174] and two-way ANOVA (analysis of variance) method [175] gave the highest performance in a recent DREAM challenge [160].

Boolean regulatory networks [135] are a special case of discrete dynamical models where the state of a node or a set of nodes exists in a binary state. The actual state of each node or set of nodes is determined by using Boolean operations on the state of other nodes in the network [153]. Boolean networks are extremely useful when amount of quantitative data is small [135, 153] but yield high number of false positives (i.e., when a given condition is satisfied while actually that is not the case) that may be reduced by using prior knowledge [176, 177]. Another bottleneck is that Boolean networks are prohibitively expensive when the number of nodes in network is large. This is due to the number of global states rising exponentially in the number of entities [135]. A method to overcome this bottleneck is to use clustering to break down the problem size. For example, Martin et al. [178] broke down a 34,000-probe microarray gene expression dataset into 23 sets of metagenes using clustering techniques. This Boolean model successfully captured the network dynamics for two different immunology microarray datasets. The dynamics of gene regulatory network can be captured using ordinary differential equations (ODEs) [155–158]. This approach has been applied to determine regulatory network for yeast [155]. The study successfully captured the regulatory network which has been characterized using experiments by molecular biologists. Reconstruction of a gene regulatory network on a genome-scale system as a dynamical model is computationally intensive [135]. A parallelizeable dynamical ODE model has been developed to address this bottleneck [179]. It reduces the computational time to *𝒪*(*N* ^2^) from time taken in other approaches which is *𝒪*(*N* ^3^) or *𝒪*(*N* ^2^log⁡*N*) [179]. Determining connections in the regulatory network for a problem of the size of the human genome, consisting of 30,000 to 35,000 genes [16, 17], will require exploring close to a billion possible connections. The dynamical ODE model has been applied to reconstruct the cardiogenic gene regulatory network of the mammalian heart [158]. A summary of methods and toolkits with their applications is presented in Table 2.

## 5. Conclusion

Big data analytics which leverages legions of disparate, structured, and unstructured data sources is going to play a vital role in how healthcare is practiced in the future. One can already see a spectrum of analytics being utilized, aiding in the decision making and performance of healthcare personnel and patients. Here we focused on three areas of interest: medical image analysis, physiological signal processing, and genomic data processing. The exponential growth of the volume of medical images forces computational scientists to come up with innovative solutions to process this large volume of data in tractable timescales. The trend of adoption of computational systems for physiological signal processing from both research and practicing medical professionals is growing steadily with the development of some very imaginative and incredible systems that help save lives. Developing a detailed model of a human being by combining physiological data and high-throughput “-omics” techniques has the potential to enhance our knowledge of disease states and help in the development of blood based diagnostic tools [20–22]. Medical image analysis, signal processing of physiological data, and integration of physiological and “-omics” data face similar challenges and opportunities in dealing with disparate structured and unstructured big data sources.

Medical image analysis covers many areas such as image acquisition, formation/reconstruction, enhancement, transmission, and compression. New technological advances have resulted in higher resolution, dimension, and availability of multimodal images which lead to the increase in accuracy of diagnosis and improvement of treatment. However, integrating medical images with different modalities or with other medical data is a potential opportunity. New analytical frameworks and methods are required to analyze these data in a clinical setting. These methods address some concerns, opportunities, and challenges such as features from images which can improve the accuracy of diagnosis and the ability to utilize disparate sources of data to increase the accuracy of diagnosis and reducing cost and improve the accuracy of processing methods such as medical image enhancement, registration, and segmentation to deliver better recommendations at the clinical level.

Although there are some very real challenges for signal processing of physiological data to deal with, given the current state of data competency and nonstandardized structure, there are opportunities in each step of the process towards providing systemic improvements within the healthcare research and practice communities. Apart from the obvious need for further research in the area of data wrangling, aggregating, and harmonizing continuous and discrete medical data formats, there is also an equal need for developing novel signal processing techniques specialized towards physiological signals. Research pertaining to mining for biomarkers and clandestine patterns within biosignals to understand and predict disease cases has shown potential in providing actionable information. However, there are opportunities for developing algorithms to address data filtering, interpolation, transformation, feature extraction, feature selection, and so forth. Furthermore, with the notoriety and improvement of machine learning algorithms, there are opportunities in improving and developing robust CDSS for clinical prediction, prescription, and diagnostics [180, 181].

Integration of physiological data and high-throughput “-omics” techniques to deliver clinical recommendations is the grand challenge for systems biologists. Although associating functional effects with changes in gene expression has progressed, the continuous increase in available genomic data and its corresponding effects of annotation of genes and errors from experiment and analytical practices make analyzing functional effect from high-throughput sequencing techniques a challenging task.

Reconstruction of networks on the genome-scale is an ill-posed problem. Robust applications have been developed for reconstruction of metabolic networks and gene regulatory networks. Limited availability of kinetic constants is a bottleneck and hence various models attempt to overcome this limitation. There is an incomplete understanding for this large-scale problem as gene regulation, effect of different network architectures, and evolutionary effects on these networks are still being analyzed [135]. To address these concerns, the combination of careful design of experiments and model development for reconstruction of networks will help in saving time and resources spent in building understanding of regulation in genome-scale networks. The opportunity of addressing the grand challenge requires close cooperation among experimentalists, computational scientists, and clinicians.

## Figures and Tables

**Table 1:** Challenges facing medical image analysis.

Challenges	Description and possible solutions
Preprocessing	Medical images suffer from different types of noise/artifacts and missing data. Noise reduction, artifact removal, missing data handling, contrast adjusting, and so forth could enhance the quality of images and increase the performance of processing methods. Employing multimodal data could be beneficial for this purpose [63–65].

Compression	Reducing the volume of data while maintaining important data such as anatomically relevant data [55, 61, 66].

Parallelization/real-time realization	Developing scalable/parallel methods and frameworks to speed up the analysis/processing [61].

Registration/mapping	Aligning consecutive slices/frames from one scan or corresponding images from different modalities [67, 68].

Sharing/security/anonymization	Integrity, privacy, and confidentiality of data must be protected [55, 69–71].

Segmentation	Delineation of anatomical structure such as vessels and bones [50, 68, 72].

Data integration/mining	Finding dependencies/patterns among multimodal data and/or the data captured at different time points in order to increase the accuracy of diagnosis, prediction, and overall performance of the system [47, 49, 52, 73].

Validation	Assessing the performance or accuracy of the system/method. Validation can be objective or subjective. For the former, annotated data is usually required [74–76].

**Table 2:** Summary of popular methods and toolkits with their applications.

Toolkit name	Category	Selected applications
Onto-Express [139, 140]	Pathway analysis	Breast cancer [141]
GoMiner [142]	Pathway analysis	Pancreatic cancer [143]
ClueGo [144]	Pathway analysis	Colorectal tumors [145]
GSEA [146]	Pathway analysis	Diabetes [147]
Pathway-Express [148]	Pathway analysis	Leukemia [149]
Recon 2 [150]	Reconstruction of metabolic networks	Drug target prediction studies [151]
Boolean methods [135, 152, 153]	Reconstruction of gene regulatory networks	Cardiac differentiation [154]
ODE models [155–158]	Reconstruction of gene regulatory networks	Cardiac development [158]
